# Short-Term Electrical Stimulation Impacts Cardiac Cell Structure and Function

**DOI:** 10.1155/term/3748093

**Published:** 2025-06-06

**Authors:** Kristen Allen, Natalie Pachter, Abigail Bandl, Haleema Qamar, Alex Ropars, Tracy A. Hookway

**Affiliations:** Department of Biomedical Engineering, Binghamton University, The State University of New York, Binghamton, New York 13902, USA

**Keywords:** cardiomyocytes, contractility, electrical stimulation, in vitro modeling, induced pluripotent stem cell, maturation

## Abstract

Induced pluripotent stem cell-derived cardiomyocytes (iPSC-CMs) are used to model cardiac development and disease. This requires a robust population of mature CMs and external stimuli to mimic the complex environment of the heart. In effort toward this maturation, previous groups have applied electrical stimulation (ES) to CMs with varying results depending on the stimulation duration, frequency, and pattern. As such, there is an uncertainty surrounding the timeline on which stimulated iPSC-CMs begin to show early signs of maturation in comparison with their nonstimulated counterparts. Here, we introduce a low-cost custom bioreactor capable of delivering tunable ES to standard 2D cell monolayers. We show that, after exposure to short-term ES, stimulated CMs express early signs of maturation compared to nonstimulated controls. Changes to contractility and protein expression indicate cellular rearrangement within cell monolayers and induction of partial maturation in response to ES. While early signs of maturation are present after 3-4 days of ES, additional cellular structures must develop to reach complete maturation. We also show that this bioreactor can electrically stimulate cardiac fibroblasts (cFBs) and may induce alignment of cFB. We have shown that our custom ES bioreactor can be easily integrated into standard in vitro cell culture platforms to induce measurable changes in both CMs and cFB, exhibiting its potential for promoting crucial CM maturation and cell alignment for cardiac tissue engineering applications.

## 1. Introduction

The characterization of cardiac physiology and disease requires models of human in vivo tissue as discoveries made in murine, bovine, or porcine models often do not translate to human subjects [1]. Because of these limitations, many groups have shifted to using in vitro models to model cardiac physiology and disease. Induced pluripotent stem cells (iPSCs) offer a robust, ethical cell source for in vitro cardiac models, but there are still several concerns regarding the reliability of these models and whether the results are translatable to in vivo pathologies [2, 3]. Ongoing research is focused on enhancing iPSC-derived in vitro models to resemble in vivo tissue.

Robust differentiation protocols have been developed to differentiate iPSCs to cardiomyocytes (CMs), the contractile muscle cells within the heart [4]. Stem cell-derived CMs are fetal in phenotype, featuring disorganized sarcomeres, a lack of transverse tubules (t-tubules), spontaneous beating, less robust calcium handling, and a flattened shape as opposed to the elongated rod shape of mature CMs [2]. Long-term culture of iPSC-CMs without additional microenvironmental cues does not result in consistent maturation within a timeline feasible for disease modeling in vitro; after 3-4 months of iPSC-CM in vitro culture, myofibril alignment and calcium handling are improved, but t-tubules are still absent [2]. Mature CMs exhibit organized sarcomeric structures, synchronized beating with neighboring cells, and a rod-like cell shape [5]. These characteristics mirror adult cells in vivo and are ideal for studying CM gene expression, structure, and ion handling.

Other groups have employed various methods to induce maturation of fetal-like iPSC-derived CMs, including electrical [6, 7], mechanical [7–9], and chemical stimulation [10, 11]. Electrical stimulation (ES) is popular due to the role of electrical signals in the in vivo cardiac system. To begin a beat, an electrical signal is sent from the SA node to the AV node between the right atrium and ventricle [12]. Upon receipt, the signal travels from the AV node through the bundle branches and subendocardial conducting network to coordinate the beat of the ventricular myocardium [13, 14]. These signals establish different cardiac rhythms, including normal sinus rhythm, arrhythmias such as ventricular tachycardia, and other pathological rhythms such as bradycardia [15]. This crucial role of electrophysiology in CM behavior in vivo motivates the exploration of how ES affects in vitro cardiac tissue models.

ES has been applied with varying results depending on the stimulation duration, frequency, and pattern [16–19]. There is uncertainty surrounding the timeline on which stimulated iPSC-derived CMs move toward maturation in comparison with their nonstimulated counterparts and when hallmarks of early maturation, such as improved contractile function, hypertrophy, changes to sarcomeric structure, and formation of t-tubules, develop. Further, cardiac fibroblasts (cFBs) are abundant in the heart and receive electrical cues alongside CMs in vivo, but little in vitro work has focused on ES of cFB. Additionally, many existing ES systems are costly and difficult to use. Here, we have developed a low-cost ES bioreactor plate lid that fits a standard 6-well plate and provides ES with tunable frequency and rhythm. The device can be easily integrated into existing cell cultures and can simultaneously provide 2 different stimulation conditions within the same plate. Using this bioreactor, we assessed a novel stimulation time point not often assessed in literature (3-4 days) and observed changes in both morphology and function in CMs and cFB upon variable frequency ES.

## 2. Methods

### 2.1. Differentiation of iPSC-CMs

Two lines of iPSCs, one genetically encoded with a GCaMP-6F calcium reporter (Gladstone Institutes, referred to as WTC cells) and one with a cardiac troponin I reporter (Allen Institutes, referred to as TNNI cells), were differentiated to CMs. iPSCs were plated at a density of 46 k cells/cm^2^ on Matrigel-coated tissue culture plates and differentiated to CMs by modulating the Wnt pathway, yielding committed and spontaneously beating CMs by Day 10 WTC [4]) or Day 14 (TNNI [20, 21]). Upon differentiation, CMs were replated for ES. Briefly, CMs were washed twice with phosphate-buffered saline (PBS), leaving the second wash on for 18 min at room temperature to aid dissociation. Cells were dissociated with trypsin (Corning) for 10 min at 37°C, then counted, and replated onto Matrigel-coated 6-well polystyrene plates at 65,000 cells/cm^2^ for low-density studies or 300–475,000 cells/cm^2^ (WTC) and 270,000 cells/cm^2^ (TNNI) for high-density studies. The replated high-density cells formed cohesive monolayers and resumed spontaneous beating within 4 days of replating. CMs were fed with RPMI/B27 with insulin (RPMI/B27+) supplemented with 1% penicillin–streptomycin (Pen/Strep) every 3 days.

### 2.2. cFB Culture

Primary adult human cFBs (Cell Applications) were thawed and seeded on tissue culture-treated 6-well polystyrene plates at 42,000 cells/cm^2^. cFBs were fed with commercially available cFB media (Cell Applications) or standard cFB media (DMEM/F12, 10% fetal bovine serum, 1% L-glutamine, 1% nonessential amino acids, 1% Pen/Strep) every 2 days until reaching confluence.

### 2.3. Custom ES Bioreactor Design

**Figure 1:**
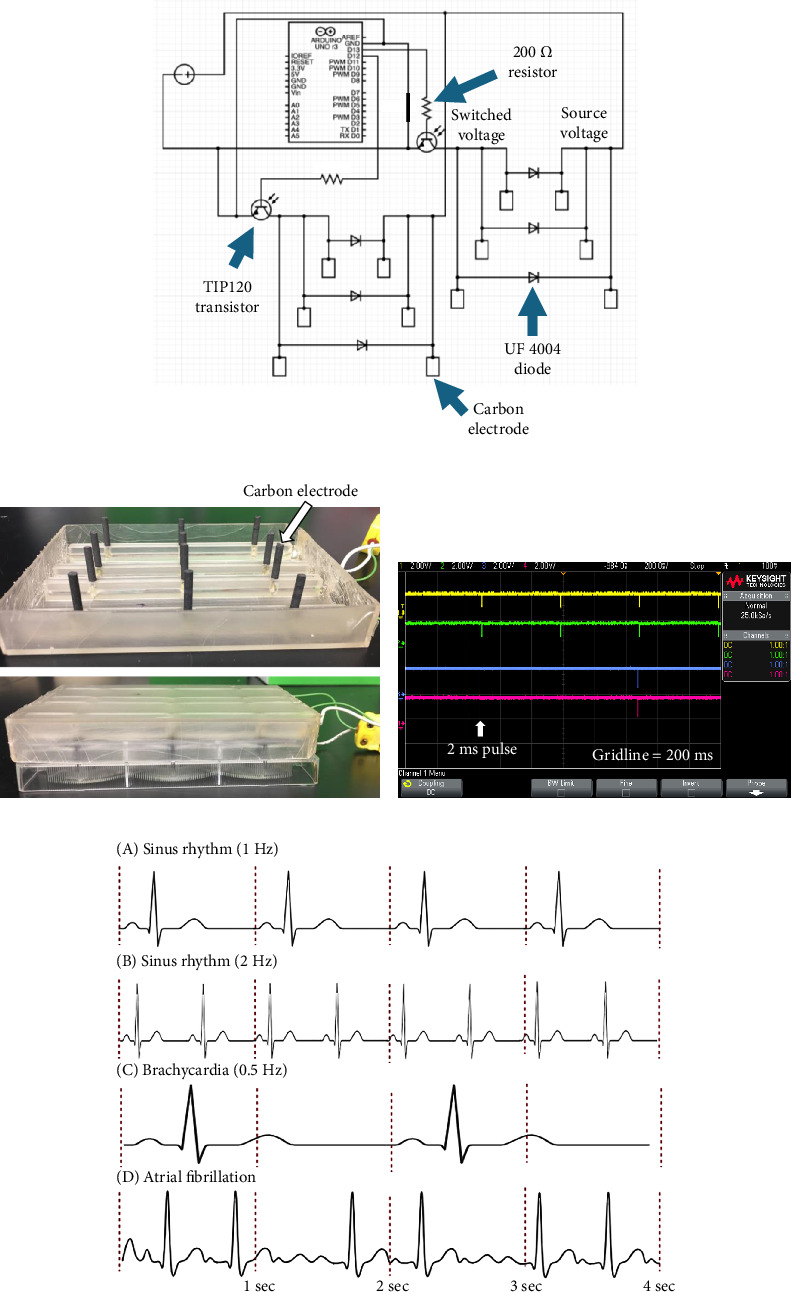
Design and verification of the custom electrical stimulation device. (a) Circuit schematic, (b) custom lid to standard 6-well cell culture plate, (c) oscilloscope reading of “switched voltage” side of UF4004 diode and corresponding electrode (yellow = 2 Hz diode, green = 2 Hz electrode, blue = 1 Hz diode, red = 1 Hz electrode), (d) physiologically relevant cardiac rhythms with the red dotted lines indicating 1 s of time.

The device consists of a 6-well plate lid 3D printed (Formlabs) in Formlabs BioMed clear resin (Figure 1(b), Supporting Figure 1); 12 rod-shaped carbon electrodes protrude from the inner surface of the lid down into the culture medium contained in the wells. The lid is designed such that two electrodes sit in each well of the 6-well plate at 25 mm apart providing bipolar stimulation of the cardiac tissue by propagating electrical signal throughout culture medium [22] at 1.15 V/cm, similar to the design featured in Mobini et al. [23]. The lid is attached to an electrical box featuring an Arduino UNO microprocessor and control circuit (Figure 1(a)). Work is done through the cell culture media when the Arduino microprocessor switches the state of the transistor, allowing current to flow between the electrodes. The circuit (Figure 1(a)) is designed with a transistor that functions as a switch. Hence, when current flows through the electrodes during stimulation, each of the electrodes has different voltage values. “Source voltage” refers to the voltage from the DC power supply that can be measured prior to work being done across the electrodes, and “switched voltage” refers to the voltage measured after work is done across the cell culture media. The “source voltage” and “switched voltage” will be equivalent when the switch (transistor) is open, but the “switched voltage” will be less than the “source voltage” when the switch is closed due to the voltage drop across the electrodes, indicating work is done across the cell culture media and stimulation is imparted to the CMs.

To impart a voltage density of 1.15 V/cm throughout the cell culture media, 2.8 V of DC power was supplied to the circuit through the power supply [24]. There was a decrease between the input voltage (2.8 V) from the power supply and the voltage measured on the “source voltage” electrode (1.6–2.0 V, corresponding to 0.64–0.8 V/cm). This is likely due to the inherent resistance in the wires and components; however, the voltage density was calculated based on the input voltage from the DC power supply.

The device is designed to run any ES pattern desired by the user via editing of the Arduino code. In this study, the device was set to provide 2 ms square monophasic pulses because this mirrors in vivo sinus rhythm, and the 2 ms stimulation is long enough to generate an excitation response from CMs [12, 25–27]. Due to resin deformation after repeated autoclaving, the device was instead sterilized in 70% ethanol for 20 min, washed with cell culture grade water, and then exposed to UV light for 30 min.

### 2.4. ES

CMs were allowed to recover for 4 days after replating and then exposed to 0 (unstimulated control), 1, and 2 Hz of ES for 3 days before contractility assessment [16]. Initial feasibility studies using this 3-day stimulation period were conducted on WTC CMs. After observing viability after 3 days of stimulation, an additional day of stimulation was added for subsequent studies using TNNI CMs. Monocultures of cFBs, once confluent, were exposed to the same ES parameters for 4 days as TNNI CMs.

### 2.5. Contractility and Beat Kinetic Assessments

Though ES of TNNI CMs was continued for 4 days as compared to 3 days on WTC CMs, contractile analysis was performed at consistent timepoints (days 0, 1, and 3) across both cell populations. On days 0, 1, and 3 of ES, ES was paused for 1 h for imaging. The cells were placed on a heated stage (Ibidi Silver Line) for the acquisition of high-quality phase videos at least 33 frames per second, with frame rate kept consistent within the same day of imaging a singular experiment (Nikon Eclipse Ti2 with equipped Andor Zyla camera). Phase videos capture contractile activity but not calcium flux and can therefore be used to assess contractile function of both WTC and TNNI CMs. As such, phase videos were analyzed here instead of GCaMP calcium flux videos to allow more comparable analyses between the 2 cell lines. Five regions of interest (ROIs), each measuring 100 × 100 μm, were captured for each of the 3 wells per condition. The videos were converted from ND2 to AVI format using a custom ImageJ macro and then processed by MUSCLEMOTION, an ImageJ Plugin used to analyze beat kinetics [28–30] (Supporting Figure 2). MUSCLEMOTION contraction profiles were manually checked to ensure all contractile peaks were captured by the software. An ROI was considered compromised if a visible artifact was present in the video or if the ROI was not beating during capture. A well, or technical replicate, was included in analysis if it retained at least 2 non-compromised ROIs. The ROIs were averaged across each technical replicate, resulting in 2-3 technical replicates per condition within a single experiment, with most technical replicates derived from an average of 4-5 ROIs. These five experiments were then pooled to the resulting 11-14 data points contained in each boxplot of the contraction data.

### 2.6. Immunostaining

After 1 day of recovery from stimulation, cells were fixed with 4% paraformaldehyde and washed 3 times with PBS. TNNI CMs were stained with Hoechst (1 : 4500) for 20 min. Images were acquired on a Nikon Ti2 Eclipse microscope with Andor Zyla camera. The green fluorescent cardiac troponin I (TNNI) expression of the CMs was measured through mean intensity using ImageJ [29, 30] and further quantified by thresholding, binarizing, and measuring the percent coverage of the green fluorescence (TNNI).

cFBs were stained with phalloidin (1 : 50) and Hoechst (1 : 4500). Actin filament alignment based on phalloidin staining was assessed with DirectionalityJ, a built-in package for ImageJ [29–32]. Only directionality measurements with a “goodness-of-fit” (GOF) value greater than 0.5 were retained for further quantitative analysis. Data below this threshold were excluded because a low GOF value indicates that the orientation measurements taken in the image were highly variable. This variability in directionality could not be reliably represented by a single directionality value, which may have skewed quantitative analysis. Each data point is representative of an ROI within a well of a single population of cFBs.

### 2.7. Statistical Analysis

All data were assessed for normality and homogeneity using the Shapiro–Wilks test and Levene's test, respectively. For data containing one variable all of the groups displayed normality and homogeneity, so one-way ANOVA was utilized to assess statistical significance. For data containing 2 variables the data were found to be homogeneous, but not normally distributed. Despite the lack of normality, 2-way ANOVA was still run to elucidate how increasing time in culture and stimulation group impacted the cells concurrently. This is considered a gold standard in the field, and 2-way ANOVA is an extremely conservative statistical test for non-normal data, more so than any nonparametric alternative [33].

## 3. Results

### 3.1. Hardware and Software Verification of ES Device

The hardware and software of the ES bioreactor were verified by oscilloscope readings taken on the “switched voltage” side of the diode and matching electrode (Figure 1(c)). This test verified that work was being done across the electrodes at a pulse width of 2 ms through the voltage drop measured on the “switched voltage” electrode. It further verified that stimulation was occurring at the correct frequency of 1 Hz and 2 Hz, and the correct voltage density of 1.15 V/cm was being imparted through the liquid, as this was the amplitude of the voltage drop seen in Figure 1(c). Finally, the equivalence of the diode and electrode measurements verified the UF4004 diode was a viable test point for what was occurring across the electrodes. Validation of this diode means users can test if the correct stimulation protocol is being run in the device during cell culture studies without compromising the sterility of the experiment.

### 3.2. Compatibility of CM Culture Is Dependent on Voltage and Seeding Density

**Figure 2:**
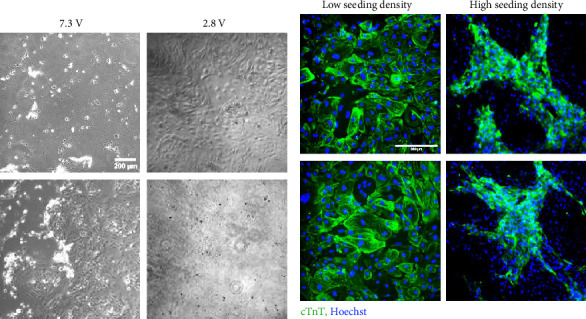
Biological verification of custom electrical stimulation device. (a) Comparison of cell attachment in response to electrical stimulation at two imparted voltages, (b) comparison of cell attachment in WTC-CM monolayer cultures at two different seeding densities in response to stimulation at 2.8 V. Scale bars = 200 μm.

2D monolayers of CMs were initially exposed to 2.92 V/cm (7.3 V from the DC power supply) or 1.15 V/cm (2.8 V from power supply) [18, 24]. Upon exposure to 2.92 V/cm, CMs experienced extensive detachment after 2 days of stimulation (Figure 2(a)). This indicated that this high voltage density was not compatible with CM monolayers on polystyrene. When the voltage density was subsequently decreased to 1.15 V/cm, CMs maintained cohesive, beating monolayers (Figure 2(a)), indicating that the lower voltage was compatible with 2D culture on polystyrene [24].

Upon confirmation of a compatible voltage density, the impact of cell density with respect to ES was further investigated. During initial feasibility studies, the need for such investigation was elucidated by the cell detachment observed directly under the carbon electrodes when the monolayers were formed from a low seeding density of 67,700 cells/cm^2^. However, after 1 Hz stimulation for 3 days at this initial seeding density, only an average of 33,970 ± 5645 cells/cm^2^ remained attached on the plate compared to an average of 38,100 ± 10,555 cells/cm^2^ in the unstimulated control. The cell layers at this low seeding density were compromised in the areas under the electrode, and the CMs did not arrange into connected, uniformly beating regions. Further feasibility studies conducted at higher seeding densities of 270,000–300,000 cells/cm^2^ yielded cohesive monolayers developed as pictured in Figure 2(b). After 1 Hz stimulation for 3 days at this higher cell seeding density, an average of 221,110 ± 16,650 cells/cm^2^ remained attached to the plate forming dense cohesive beating patterns within the syncytium compared to an average of 198,975 ± 26,470 cells/cm^2^ in the unstimulated control. Additionally, the monolayers formed from the higher seeding density did not detach in the regions directly below the carbon electrodes. Therefore, subsequent studies with CM monolayers were conducted at 270,000–300,000 cells/cm^2^.

### 3.3. cFB Culture Is Compatible With ES Device

**Figure 3:**
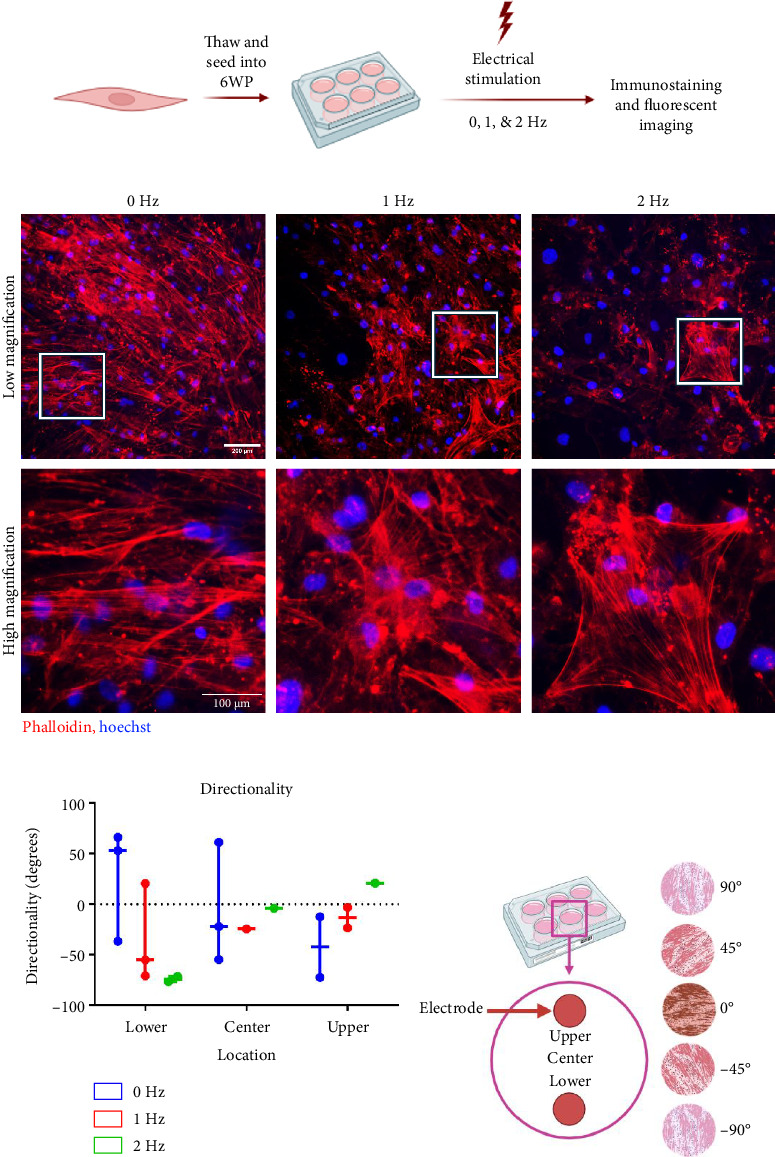
Custom electrical stimulation device is compatible with cardiac fibroblasts. (a) Electrical stimulation experimental workflow on cFBs, (b) fluorescent images of phalloidin and Hoechst staining of cFBs exposed to stimulation at low (top row, scale bar = 200 μm) and high (bottom row, scale bar = 100 μm) magnification. White frames on low magnification images indicate regions shown in high magnification images. (c) Directional orientation of cFBs sorted by location in the well, (d) schematic of the well locations and orientations.

We next aimed to elucidate how ES impacts cFBs, since these cells are also abundant in the native heart and are important for propagation of electrical signal. Given the effect of cell density observed with CMs, 2D cFBs monocultures were seeded and grown to confluence prior to initiating ES. Upon exposure to 1 Hz of stimulation for 4 days at 1.15 V/cm, cFBs remained attached and exhibited robust actin filaments as shown by phalloidin staining (Figure 3(b)). There was a slight decrease in actin filament expression in the 2 Hz condition, but cells maintained a complete monolayer in all conditions, indicating the compatibility of the stimulation device with both CMs and cFBs.

Directionality of actin filaments was used to determine if cFBs reorganized their structural arrangement in response to ES at the same frequency and voltage densities as CMs. After 4 days of 0, 1, and 2 Hz stimulation, no discernible trend in cFB alignment was seen at the center of the wells; however, the greatest rearrangement in directionality was observed in the upper and lower areas closest to the electrodes (Figures 3(c) and 3(d)). Although not significant, the stimulated groups, particularly the 2 Hz group, exhibited greater alignment near the electrodes. This can be seen by the 2 Hz group angled at −80° by the lower electrode, whereas cFB naturally arranged in the positive direction in the lower region of the 0 Hz condition. Likewise, the 2 Hz group oriented in the positive direction near the upper electrode when the 0 Hz group naturally arranged on the plate in the negative direction. These observations suggest that the directionality of cFB near the electrodes diverges from the directionality of the nonstimulated group. This response indicates that cFBs are compatible with the device and that the device may be able to induce cellular rearrangement.

### 3.4. CMs Exhibit Structural Changes Under ES

**Figure 4:**
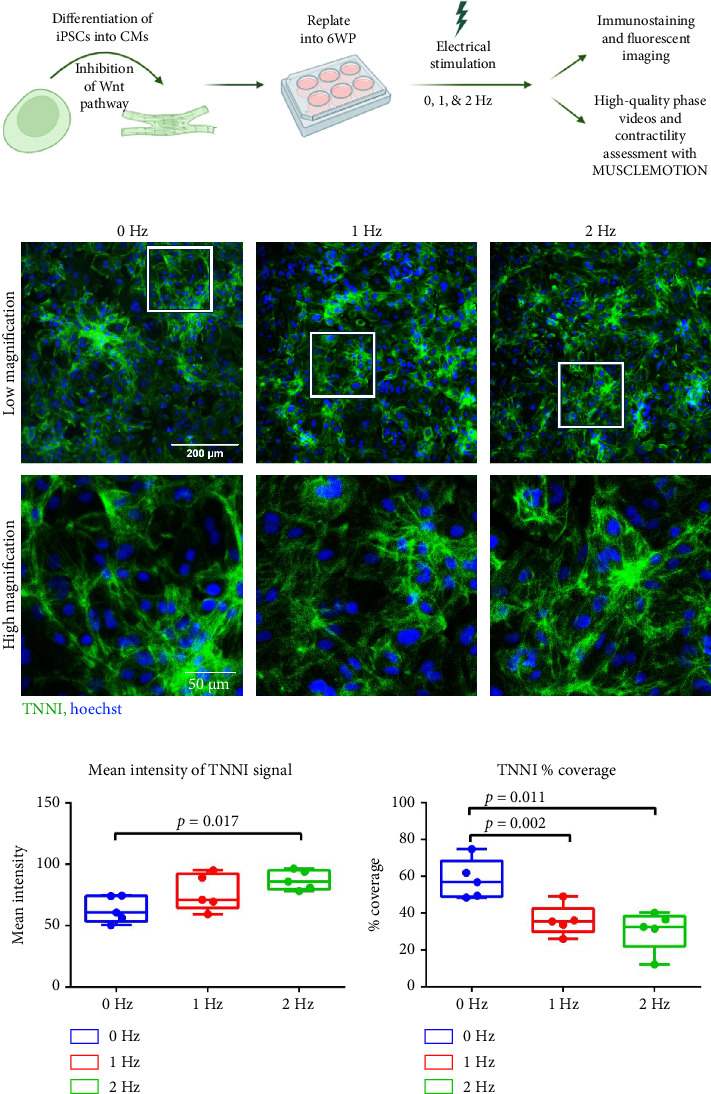
Robust monolayers of cardiomyocytes undergo structural changes after exposure to 4 days of electrical stimulation. (a) Electrical stimulation experimental workflow on TNNI CMs, (b) fluorescent images of TNNI expression and Hoechst staining of CM at low (top row, scale bar = 200 μm) and high (bottom row, scale bar = 50 μm) magnification. White frames on low magnification images indicate enlarged regions shown in high magnification images. Expression of TNNI measured by (c) mean intensity in a fluorescent image and (d) percent coverage.

After 4 days of ES, most CMs remained attached to the polystyrene plate (Figure 4(b)). The comparison of the mean intensity of fluorescently-tagged TNNI across the stimulation groups shows significantly greater TNNI intensity in the 2 Hz group as compared to the 0 Hz group, indicating a stronger expression of sarcomeric proteins in the stimulated CMs (Figure 4(c)). In contrast, the percent coverage of TNNI expression significantly decreased in the stimulated groups compared to the 0 Hz control (Figure 4(d)), suggesting fewer cells in the 1 Hz and 2 Hz conditions. Taken together, these measures indicate that although ES may have resulted in fewer CMs in 1 Hz and 2 Hz conditions, ES may also have resulted in increased cell organization.

### 3.5. iPSC-Derived CMs Express Changes in Contractility After Exposure to Short-Term ES

**Figure 5:**
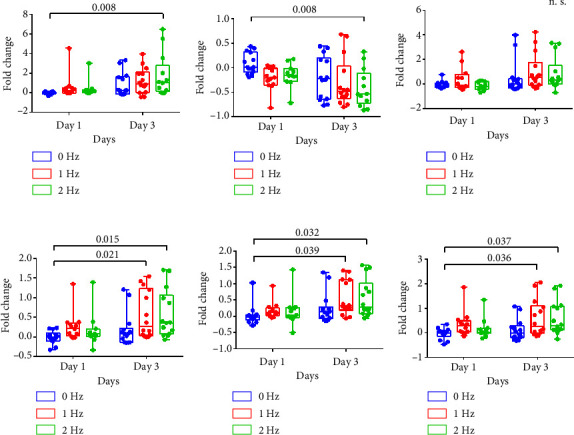
Changes in CM contractile parameters in response to time in culture and stimulation frequency. Fold change in (a) peak-to-peak time, (b) beat frequency (beats per minute), (c) contraction amplitude, (d) contraction duration, (e) time-to-peak, and (f) relaxation time as compared to Day 0 values from 2D monolayer cultures of WTC- and TNNI-derived cardiomyocytes under electrical stimulation at 0, 1, and 2 Hz for 3-4 days “n.s.” indicates no significance.

To investigate the functional consequences of short-term ES on iPSC-CMs, contractility assessment was conducted on five independent populations of CMs with pooled results shown in Figure 5. After exposure to 3 days of 2 Hz stimulation, changes in beat duration and frequency were observed. This is highlighted by a significant decreases in beat frequency (Figures 5(a) and 5(b)), and increases in contraction duration (Figure 5(d)), time-to-peak (Figure 5(e)), and relaxation time (Figure 5(f)) of the 2 Hz group on Day 3 as compared to Day 1. Contraction amplitude (representing beat strength) was not affected by ES (Figure 5(c)). The significant contractile changes indicate that the combination of increased stimulation frequency and increased duration in culture induced functional changes in CMs. These changes included a reduced beat frequency and prolonged contraction. While some literature suggests that decreased contraction duration is indicative of CM maturation [34], there is evidence that prolonged action potential duration and decreased beat rate are more characteristic of early CM maturity [18, 35]. As such, reduced beat frequency and prolonged contractions observed in our stimulated CMs may be nascent signs of maturation. These changes in contractility were also consistent regardless of location within the well (Supporting Figure 3).

## 4. Discussion

In this study, we designed a custom ES bioreactor used to provide different 2D cardiac cell populations to varied stimulation frequencies. Similar to commercial devices, utilizing a standard 6-well plate format allows for seeding either monocultures, as described in this study, or the potential for coculture experiments with combinations of additional cardiac cells. In addition to easy integration into existing cell cultures, this also circumvents issues such as leakage or media transfer between wells that may be present in other fabricated devices that use custom chamber systems. The device described in this study is compatible with sterile cell culture and can also be expanded to other well sizes, with adjustments to the imparted voltage based on the distance between the electrodes. Additionally, unlike commercially available devices, this device offers flexibility to the user because of the open-source Arduino programming utilized in its design and the ability to easily reprogram the device at any time. This gives users the ability to constantly upgrade the functionality of the device, and to tune the frequency and rhythm of stimulation to represent various rhythms such as bradycardia, ventricular tachycardia, or arrhythmias such as ventricular fibrillation. The device presented here is also inexpensive (∼$350), making it more accessible to laboratories of any scale with access to a 3D printer.

There are, however, limitations to the device presented. It is likely that the voltage density is not consistent throughout the well, meaning that the cells receive varied stimulation intensity based on location [6]. This may be why increased cFB rearrangement was observed closer to the electrodes as compared to the center of the well (Figure 3(c)) and why detachment was observed under electrodes when seeding density was not high enough. However, voltage density variation can be accounted for in the analysis phase by averaging several ROIs across the well, ensuring that this device still accomplishes the ultimate goal of applying controlled stimulation to cardiac cell cultures as a method of promoting maturation. Further, no changes in CM morphology or contractile function between different locations within the well were observed across the five experiments conducted (Supporting Figure 3), indicating that overall effects of ES are consistent within wells.

Although the device is made of autoclavable materials, the resin exhibits peeling and warping in response to repeated heat and pressure cycles, which may compromise the lid's fit onto the 6-well plate and therefore sterility. Other materials, such as polycarbonate or polypropylene, that may be more compatible with repeated autoclaving will be considered for future generations of the device. Overall, the benefits of the device, including customizability and cost-effectivity, outweigh the limitations of variation in the exposed CMs and a limit on the number of autoclave cycles the device can sustain, and these limitations can be addressed in further iterations of the device's design and fabrication. The data presented indicate the device is effective at inducing a certain degree of maturation, and further work will allow for more advanced maturation of CMs.

In this study, the bioreactor was used to assess changes in cellular structure and function in response to short-term ES, modeling normal sinus rhythm at 60 and 120 beats per minute (BPM). CMs exposed to ES experienced a decrease in beat frequency and an increase in the duration of the beat. This change in behavior in response to ES may be indicative of changes to the arrangement of the CMs. One possible explanation for the change in beat pattern and the higher expression of TNNI is an alignment of the CMs in response to ES, leading to sections of the monolayer with a denser concentration of cells than others [36]. This would lead to higher expression of TNNI and changes in contractility parameters in those denser pockets of cells. Further, cell alignment and denser populations in a region would allow for coupling between cells in electric function, leading to the stronger contraction seen in the upward trend of contraction amplitude and the significant changes in contractile parameters, such as peak-to-peak time, contraction duration, time-to-peak, and relaxation time [36]. Therefore, it is possible that ES induced heterogeneity in the organization of the CM monolayer, leading to changes in TNNI expression and contractility.

Another possible explanation for this change in contractility is that the CMs experienced hypertrophy in response to ES. These beat kinetic changes are consistent with previous literature characterizing iPSC-derived CM behavior after experiencing healthy physiological hypertrophy [17, 37, 38]. Further, hypertrophy would also explain why the number of CMs decreased in the stimulated groups, as indicated by the significant decreases in the percent coverage of TNNI + cells, while the mean intensity expression of TNNI increased in the 2 Hz stimulation group (Figures 4(c) and 4(d)). While some cells detached in response to ES, the cells that remained attached could have experienced hypertrophy, increasing the expression of TNNI via sarcomere expansion.

Hypertrophic CMs have a greater distance between sarcomeres and the calcium-containing sarcoplasmic reticulum [39]. While adult CMs have t-tubules to facilitate calcium transport across this distance, fetal-like iPSC-derived CMs do not [40, 41]. As such, CM hypertrophy without t-tubule formation can delay intracellular calcium delivery and decrease beat frequency [41]. The significant differences shown in Figure 5 may suggest a partial maturation state of our stimulated CMs featuring hypertrophy, but not t-tubule formation. Notably, beat frequency can vary naturally in *in vitro* cultures; however, ES may alter spontaneous beat rate, making beat frequency a useful metric for stimulated cells.

The partial maturation of CMs in response to short-term ES captures a novel time point that is often overlooked in the literature; however, it also highlights the question of when full maturation occurs in response to ES. Extending the ES protocol in stages would also allow for identification of the time point at which cells experience t-tubule formation, which would be captured by an increase in beat frequency and through transmission electron microscopy [19, 39, 42]. Further, the formation of t-tubules may also be supported through the introduction of cFBs in coculture with CMs [40]. cFBs communicate through paracrine biochemical cues and electrical signaling to CMs to enhance cardiac activity [43, 44]. For example, the paracrine signaling among cFB and CMs using angiotensin II can directly lead to hypertrophy within CMs [43]. Further, cFBs can aid in propagating electrical signal between CMs using gap junctions, such as connexin 43, which provides convincing evidence that cFB would enhance the maturation of CMs when exposed to electrical signal [43, 44]. The foundation for further study has been laid in this work because cFBs remained attached to the culture plate using the custom stimulator lid (Figure 3(b)) and reorganized in response to ES (Figure 3(c)). This means further investigation is required into whether iPSC-derived CMs would mature faster when exposed to ES in a CM-cFB coculture. The next step in enhancing in vitro modeling is to develop a robust pipeline for the availability of robust, mature, and pure populations of CMs; ES, in combination with coculture and CM purification protocols, will likely play a key role in accomplishing this.

## 5. Conclusions

We have developed a custom ES device that can be easily incorporated into standard 2D cell cultures to provide tunable ES. Short-term ES resulted in reorganization and partial maturation of CMs and potential reorientation of CFs with respect to the electrodes. With further optimization, this device will aid maturation of iPSC-CMs for more relevant cardiac disease models.

## Data Availability

The data from this study are available upon reasonable request.

## Supporting Information

Supporting Information 1 Supporting Figure 1: Dimensions of custom cell culture lid used for ES experiments.

Supporting Information 2 Supporting Figure 2: Parameters of MUSCLEMOTION indicating CM contractile function. Visual depictions of (a) peak-to-peak time, (b) beat frequency (beats per minute), (c) contraction amplitude, (d) contraction duration, (e) time-to-peak, and (f) relaxation time placed on a representative contraction profile generated with MUSCLEMOTION. (Electrophysiological trace representing beat frequency designed in BioRender).

Supporting Information 3 Supporting Figure 3: (a), (b), (c) fluorescent images of cTnT expression displaying sarcomeric morphology in WTC-CMs, contractility data of (d) peak-to-peak time and (e) time-to-peak collected for the 1 Hz stimulation group of WTC-CMs at different locations within the well. Top and bottom regions are nearest to the electrodes.

Supporting Information 4 Supporting Video: High-quality phase video of beating WTC-CMs after 3 days of ES analyzed by MUSCLEMOTION to quantify contractile behavior.
