# Supplementary figures and images for: Short-Term Electrical Stimulation Impacts Cardiac Cell Structure and Function
Source: J Tissue Eng Regen Med. 2025 Jun 6;2025:3748093. doi: 10.1155/term/3748093 (PMC12165760; doi:10.1155/term/3748093)

## Slide 1
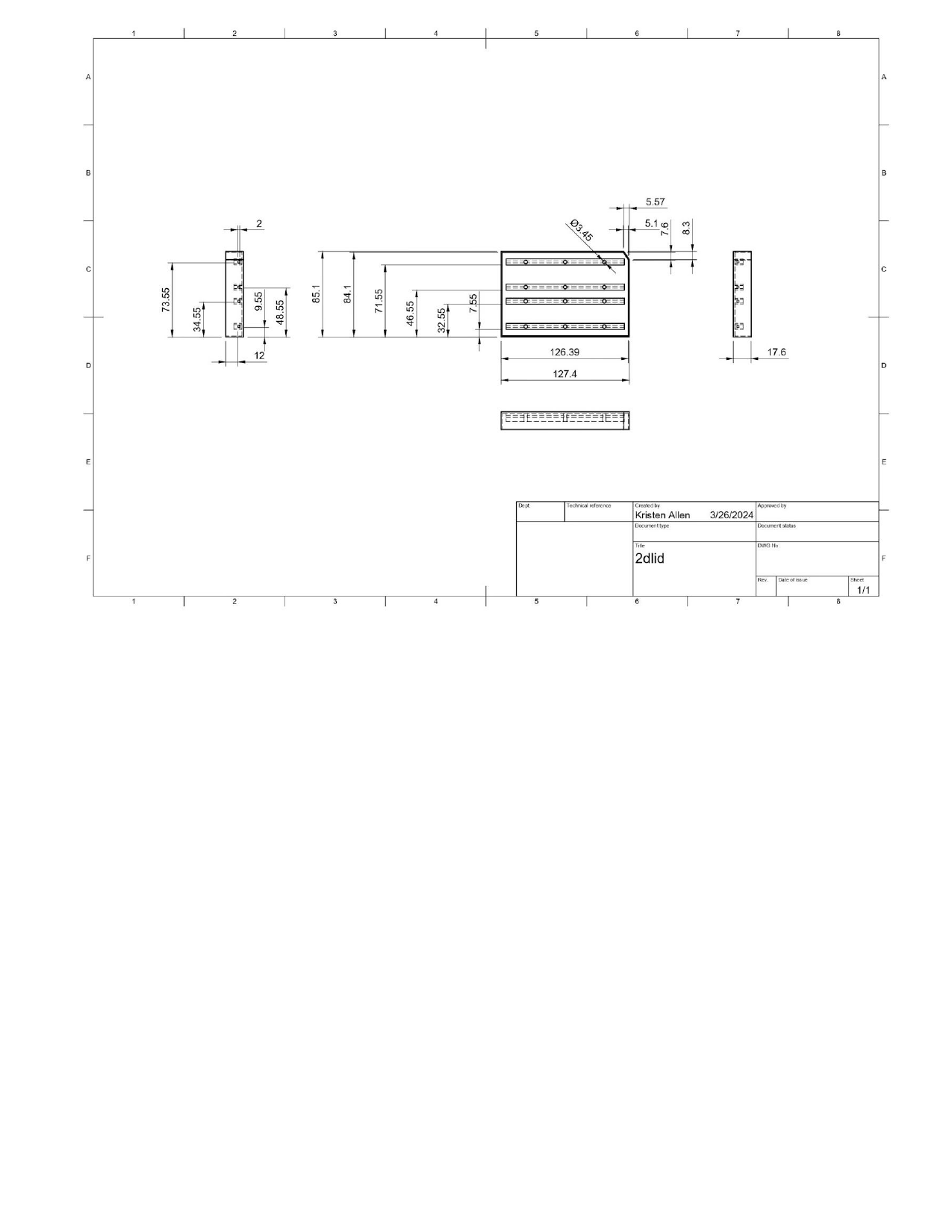

Supplement: Supporting Information 1 — Supporting Figure 1: Dimensions of custom cell culture lid used for ES experiments. [file 3748093.f1.pptx]

## Slide 1
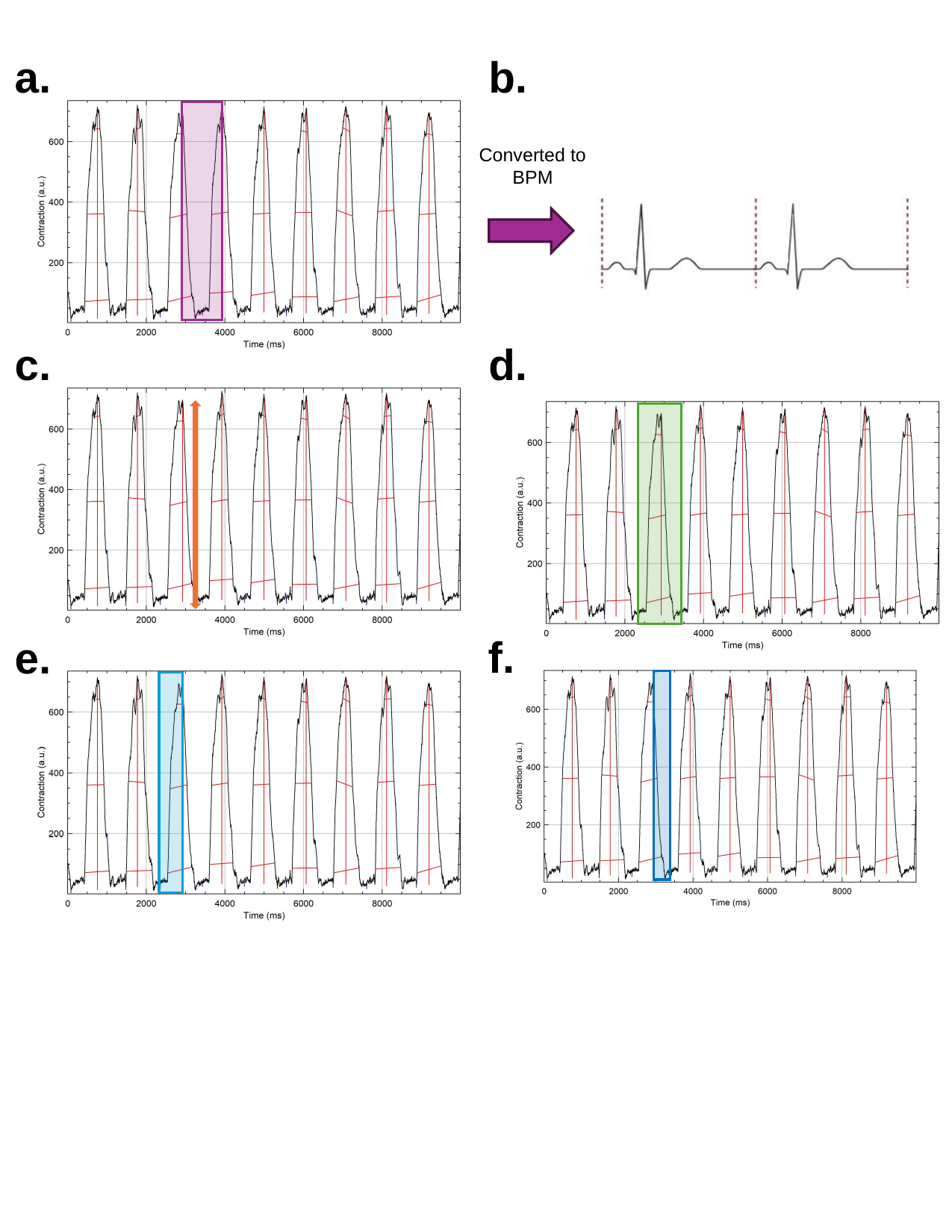

a.
b.
Converted to BPM
c.
d.
f.
e.

Supplement: Supporting Information 2 — Supporting Figure 2: Parameters of MUSCLEMOTION indicating CM contractile function. Visual depictions of (a) peak-to-peak time, (b) beat frequency (beats per minute), (c) contraction amplitude, (d) contraction duration, (e) time-to-peak, and (f) relaxation time placed on a representative contraction profile generated with MUSCLEMOTION. (Electrophysiological trace representing beat frequency designed in BioRender). [file 3748093.f2.pptx]

## Slide 1
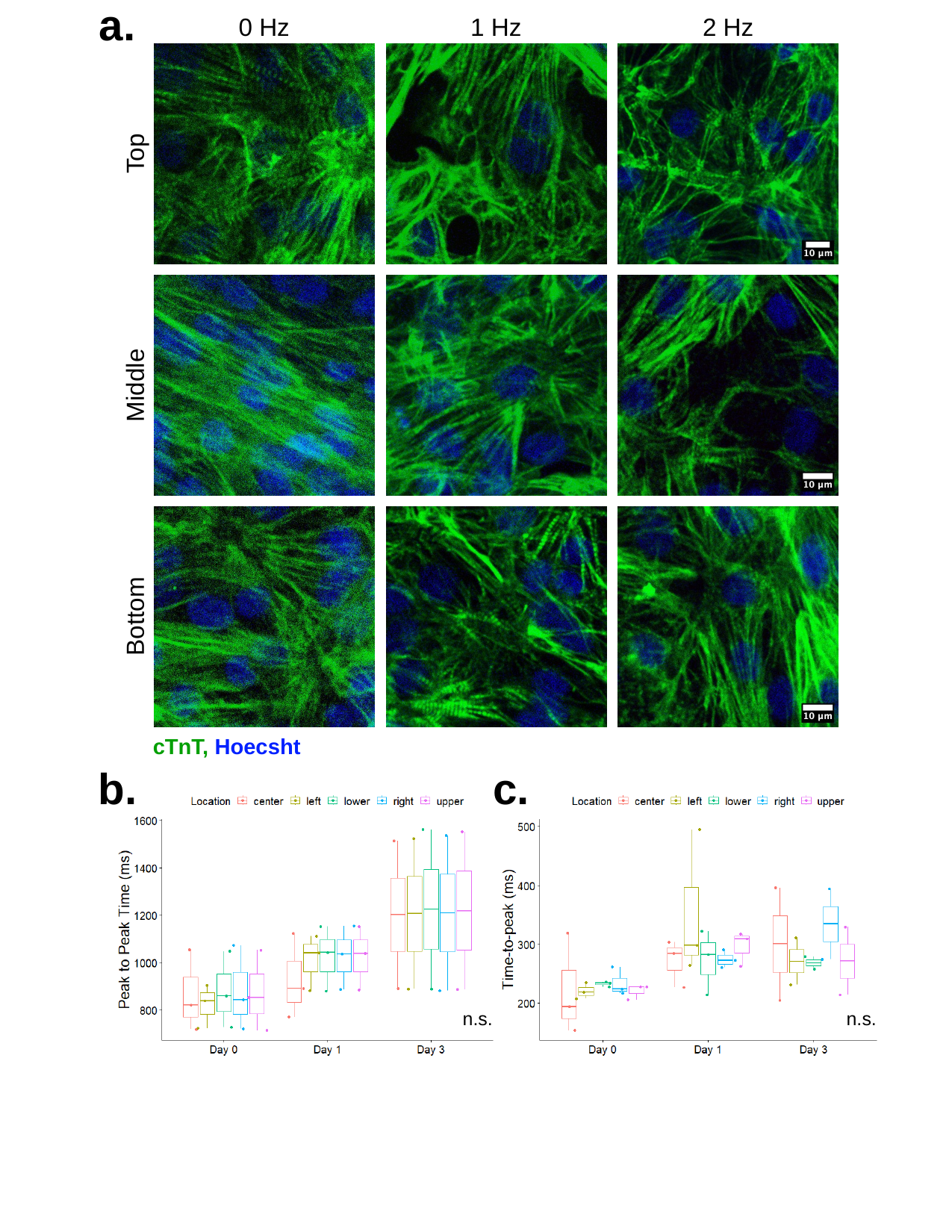

a.
0 Hz
1 Hz
2 Hz
Top
Middle
Bottom
cTnT, Hoecsht
b.
c.
n.s.
n.s.

Supplement: Supporting Information 3 — Supporting Figure 3: (a), (b), (c) fluorescent images of cTnT expression displaying sarcomeric morphology in WTC-CMs, contractility data of (d) peak-to-peak time and (e) time-to-peak collected for the 1 Hz stimulation group of WTC-CMs at different locations within the well. Top and bottom regions are nearest to the electrodes. [file 3748093.f3.pptx]
